# Interleukin-6 in peripheral blood and inflammatory sites in Behçet's disease

**DOI:** 10.1155/S0962935192000437

**Published:** 1992

**Authors:** K. Hamzaoui, A. Hamzaoui, A. Kahan, M. Hamza, A. Chabbou, Kh. Ayed

**Affiliations:** 1 Faculté de Médecine de Tunis 9 Rue Z. Essafi Tunis 1007 Tunisia; 2 INSERM U. 283 Hôpital Cochin Paris France

## Abstract

Interleukin-6, a potent pro-inflammatory cytokine, might be involved in Behçet's disease (BD) pathological pathways. We investigated IL-6 levels in sera and synovial fluids collected from BD patients. The IL-6 production was also studied in vivo, by measuring its activity in culture supernatants of PBMC and alveolar macrophages, stimulated or not with LPS. The patients with BD were compared to RA patients and healthy controls. High IL-6 levels were observed in sera, synovial fluid and LPS stimulated PBMC supernatants, from active BD patients, similar to those of RA patients. Alveolar macrophages production of IL-6 was significantly elevated in two active BD patients with an interstitial pneumonia, when compared to controls. These elevated levels of IL-6 suggest its involvement in the inflammatory sites of BD, which may be related to the progression of the acute lesions, at least in the joints and in the lungs.


Research Paper

Mediators of Inflammation, 1, 281–285 (1992)

### Interleukin-6 in peripheral blood and inflammatory sites in Behcet's disease

K. Hamzaoui,[1,CA] A. Hamzaoui,[1] A. Kahan,[2] M. Hamza,[1] A. Chabbou[1] and Kh. Ayed[1]

[1]Faculté de Médecine de Tunis, 9, Rue Z. Essafi 1007 Tunis, Tunisia;

[2]INSERM U. 283, Hôpital Cochin, Paris, France

[CA] Corresponding Author

**INTERLEUKIN-6, a potent pro-inflammatory cytokine, might be involved in Behçet's disease (BD) pathological pathways. We investigated IL-6 levels in sera and synovial fluids collected from BD patients. The IL-6 production was also studied *in vivo*, by measuring its activity in culture supernatants of PBMC and alveolar macrophages, stimulated or not with LPS. The patients with BD were compared to RA patients and healthy controls. High IL-6 levels were observed in sera, synovial fluid and LPS-stimulated PBMC supernatants, from active BD patients, similar to those of RA patients. Alveolar macrophages production of IL-6 was significantly elevated in two active BD patients with an interstitial pneumonia, when compared to controls. These elevated levels of IL-6 suggest its involvement in the inflammatory sites of BD, which may be related to the progression of the acute lesions, at least in the joints and in the lungs.**

**Key words:** Behçet's disease, Inflammatory sites, Interleukin-6

### Introduction

Behçet's disease (BD) is a multisystemic disease, mainly observed in Mediterranean areas and in Japan; it is characterized by oral and genital ulcerations, associated with ocular and skin lesions, arthralgia, neurological involvement, and pulmonary manifestations.

Elevated levels of interleukin-1 (IL-1), tumour necrosis factor-α (TNF-α),[1] soluble interleukin-2 receptor[2] and gamma-interferon[3–5] have been observed in patients with active Behçet's disease (BD), suggesting the involvement of these cytokines in the inflammatory process.

Among the immunological factors that have been recently involved in the inflammatory manifestations, interleukin-6 (IL-6) plays a prominent role.[6] IL-6 has pleitropic biological effects, including human T-cell activation,[7] and B-cell proliferation and differentiation.[8]

The aim of our study was to evaluate in BD patients IL-6 level in sera and IL-6 production by blood mononuclear cells (PBMC) into culture supernatants. Moreover, IL-6 was quantified in two local inflammatory sites: synovial fluid and culture supernatants of alveolar macrophages obtained by bronchoalveolar lavage.

### Materials and Methods

*Patients:* Thirty patients with BD, who fulfilled the criteria proposed by the International Study Group for Behçet's Disease[9] were studied, 28 men and two women, with a mean (±SEM) age of 34 ± 3 years. Twenty patients had active disease, and ten inactive disease. The clinical characteristics and the treatment of active BD patients are shown in Table 1. Two patients with active BD had also pulmonary manifestations: a chronic cough associated to interstitial shadows on the chest roentgenogram.[10] These patients received no treatment.

Six patients with inactive BD received thalidomide therapy for 6 to 38 months with an average of 20 months,[11] the other four patients with inactive disease received corticosteroids.

Ten patients with rheumatoid arthritis (RA), meeting the Arthritis Rheumatism Association criteria, were studied during acute flares of synovitis. All RA patients were on standard therapy including nonsteroidal anti-inflammatory drugs. Most patients were female, with a mean (±SEM) age of 47 ± 12 years.

Twenty healthy age-and-sex-matched subjects were studied as controls.

*Serum and synovial fluid:* Serum samples were collected from all patients and control subjects. Synovial fluid was obtained from the ten RA patients and ten active BD patients with arthritis. Freshly aspirated synovial fluids were collected into heparinized tubes (10 U $ml^{-1}$), treated with 1.5 U $ml^{-1}$ hyaluronidase for 30 min at 37°C, centrifuged at 1 200 × *g* to remove cellular debris and stored at −20°C until use.

*Peripheral blood mononuclear cells (PBMC):* IL-6 production by PBMC was studied in active BD (ten cases), inactive BD (ten cases), RA patients (ten cases) and ten healthy controls. PBMC were separated by

© 1992 Rapid Communications of Oxford Ltd

**Table 1**. Clinical characteristics of active BD patients

| Number | Age (years) | Sex | Oral ulcers | Genital ulcers | Arthritis | Uveitis | Treatment |
|---|---|---|---|---|---|---|---|
| 1 | 32 | M | + | + | + | + | CS |
| 2 | 45 | M | + | + | + | + | NT |
| 3 | 34 | M | + | + | − | + | NT |
| 4 | 27 | M | + | − | + | + | NT |
| 5 | 38 | F | + | + | − | + | NT |
| 6 | 40 | M | + | + | − | + | NT |
| 7 | 37 | M | − | + | + | + | NT |
| 8 | 26 | M | + | + | − | + | CS |
| 9 | 33 | F | + | + | + | + | NT |
| 10 | 35 | M | + | + | + | + | NT |
| 11 | 36 | M | + | + | + | + | CS |
| 12 | 48 | M | + | − | + | + | NT |
| 13 | 43 | M | + | + | + | + | NT |
| 14 | 24 | M | + | + | + | − | NT |
| 15 | 29 | M | + | + | + | + | NT |
| 16 | 45 | M | + | + | − | + | CS |
| 17 | 37 | M | + | + | + | + | NT |
| 18 | 36 | M | + | + | + | + | NT |
| 19 | 45 | M | + | + | − | + | NT |
| 20 | 22 | M | + | + | + | + | NT |

+ or − = with or without symptoms; NT = without treatment, CS = daily 0.5 mg $kg^{-1}$ prednisone standard Ficoll–Hypaque density gradient centrifugation. PBMC ($5 \times 10^6$ $ml^{-1}$) were resuspended in RPMI 1640 medium (Gibco, Grand Island, NY) supplemented with 20 $\mu$g gentamycin, 5% AB-positive serum and 2 mmol $l^{-1}$ L-glutamine. The final cellular suspension of PBMC contained 20% monocytes. Cells were cultured at a concentration of $10^6$ $ml^{-1}$, for 24 h in humidified air containing 5% $CO_2$ at 37°C, in the presence or absence of 20 $\mu$g $ml^{-1}$ of *Escherichia coli* lipopolysaccharide (LPS; Sigma, St Louis, MO). Supernatants were collected after 24 h of culture.

*Bronchoalveolar lavage:* Bronchoalveolar lavage (BAL) was performed in two patients with active BD having a chronic cough associated to interstitial shadows on the chest roentgenogram, and five control subjects, using a fibre-optic bronchoscope. The right middle lobe was lavaged with 50 ml aliquots of sterile 0.9% sodium chloride to a total of 200 ml. The lavage fluid was gently aspirated after each aliquot and collected into a sterile, siliconized glass bottle and maintained at 4°C. The lavage fluid was filtered through a coarse gauze and centrifuged at $480 \times g$ at 4°C. The cell pellet was then washed twice with RPMI 1640 medium. Alveolar macrophages (AM) were enriched by adherence to plastic (Costar, Cambridge, MA) for 24 h at 37°C and 5% $CO_2$. The adherent cells were collected and the percentage of AM ranged between 90 and 96%. The AM were placed into the culture medium at a density of $10^6$ AM/ml, and assayed for IL-6 production with or without LPS stimulation. After 24 h, AM culture supernatants were collected.

*Assessment of IL-6 activity:* IL-6 activity was tested using an IL-6-dependent mouse hybridoma 7TD1, cultivated in flat-bottomed microtitre plates containing 2 000 cells/well in the presence of serial dilutions of supernatants or sera. After 4 days of culture, the number of surviving cells was determined by a colorimetric assay as reported by Van Damme *et al.*[12] Hybridoma growth factor/IL-6 activity was expressed in U $ml^{-1}$, defined as the dilution giving half the maximal proliferation of 7TD1 cells. One unit corresponds to approximately 5 pg $ml^{-1}$ of IL-6. 7TD1 cells respond neither to IL1, TNF$\alpha$, IL2, IFN$\alpha$ and $\gamma$, nor to any of the known colony-stimulating factors other than IL-6. Biological activity of IL-6 samples was completely neutralized by adding monospecific rabbit polyclonal anti-recombinant human IL-6 antibodies to test samples. To eliminate inhibitory effects present in undiluted sera and to determine the lower limit of detection of the assay, IL-6 activity in sera was measured as such and by adding 100 U of recombinant cytokine. The standard used in these assays was r-IL-6 (Genzyme, Boston, MA). IL-6 activity in experimental samples was tested at least three times.

*Statistical analysis:* Data were analysed with a Stat Work program on a Macintosh Classic computer. All results are expressed as (mean ± SEM). Comparisons of mean values were performed with the unpaired Student's *t*-test. Non parametric comparisons were conducted with the Mann–Whitney U-Test, using Cricket Stat Work.

### Results

*IL-6 in serum:* Low levels of IL-6 activity could be detected in the serum from all healthy individuals tested (4.5 ± 1.9 U ml$^{-1}$). In patients with active BD, the values were significantly increased (19.8 ± 9 U ml$^{-1}$) (Fig. 1) compared to control subjects ($p < 0.001$): 16 patients with active BD stage and without treatment had increased levels of IL-6 (≥12 U ml$^{-1}$). The four other active BD patients receiving steroid therapy had low levels of IL-6 (<6 U ml$^{-1}$). Patients with inactive disease had similar IL-6 levels as controls (5.3 ± 2.3 U ml$^{-1}$). Patients with RA had also increased seric IL-6 levels (16.7 ± 3.2 U ml$^{-1}$; $p < 0.01$) when compared to healthy controls. No statistical difference of seric IL-6 levels between active BD and RA patients was observed.

*IL-6 in PMBC culture supernatants:* IL-6 was undetectable in monocytes depleted T-cell cultures either from BD patients, RA patients or healthy controls.

Spontaneous IL-6 production was detected in active BD patients (898 ± 78.2 U ml$^{-1}$), in inactive BD patients (272 ± 71.4 U ml$^{-1}$), in RA patients (717 ± 176.5 U ml$^{-1}$) and in healthy controls (196 ± 31.7 U ml$^{-1}$). Spontaneous IL-6 production was significantly increased in active BD patients and in RA patients (Fig. 2) when compared to inactive BD and control subjects ($p < 0.01$). There was no difference in spontaneous IL-6 production between patients with inactive BD and healthy controls.

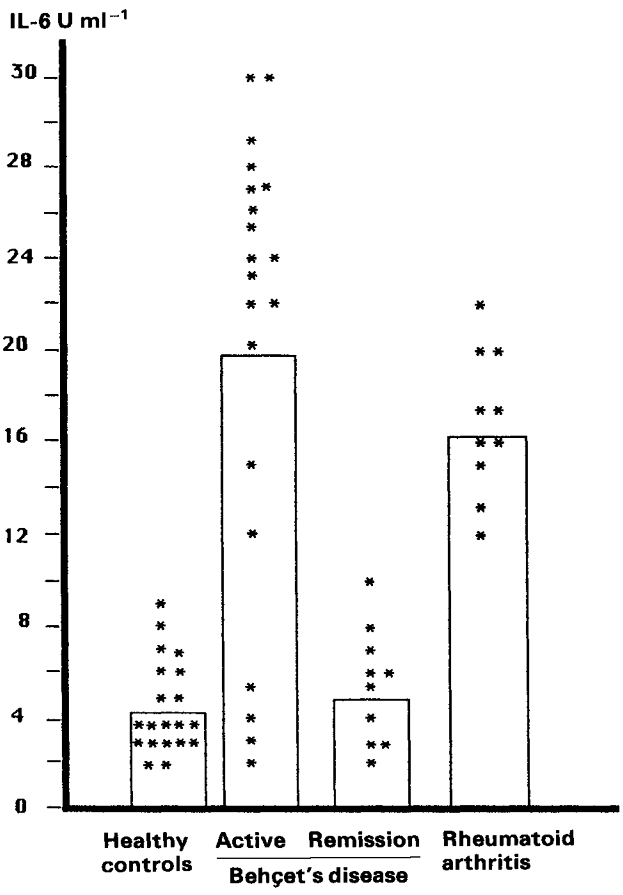

FIG. 1. Serum levels of interleukin-6 (IL-6) in healthy controls (4.5 ± 1.9 U ml$^{-1}$), in patients with active Behçet's disease (19.8 ± 9 U ml$^{-1}$), in patients with inactive Behçet's disease (5.3 ± 2.3 U ml$^{-1}$) and in patients with Rheumatoid Arthritis (16.7 ± 3.2 U ml$^{-1}$).

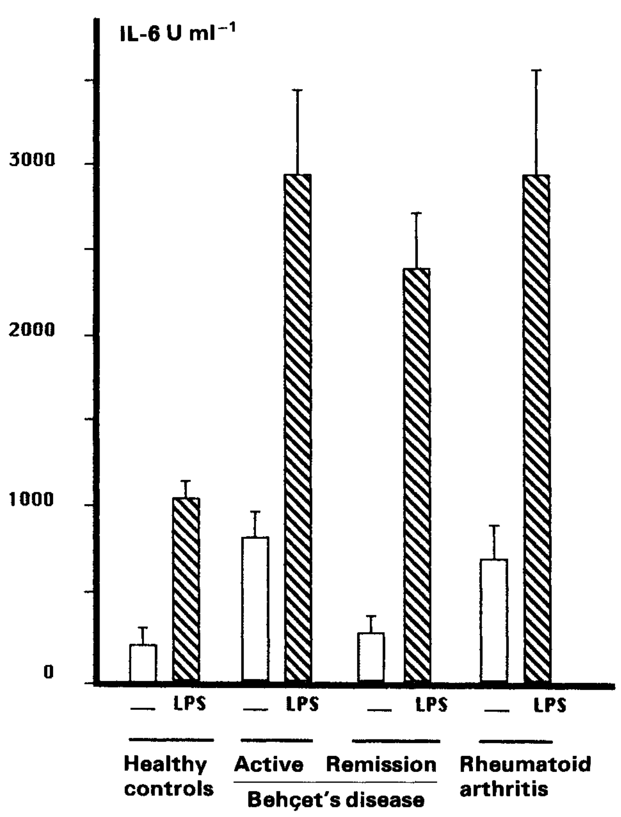

FIG. 2. Spontaneous and LPS-induced production of IL-6 (U ml$^{-1}$) by PBMC from patients with active and inactive Behçet's disease, patients with rheumatoid arthritis and from healthy controls.

After LPS stimulation IL-6 production was significantly increased ($p < 0.01$) in patients with active BD (2980 ± 518 U ml$^{-1}$), in inactive BD patients (2390 ± 310 U ml$^{-1}$), and in RA patients (2950 ± 873 U ml$^{-1}$) (Fig. 2) as compared with healthy controls (1100 ± 117 U ml$^{-1}$).

*IL-6 in synovial fluid:* IL-6 was detected in all synovial fluid samples tested. IL-6 levels were similar in joint fluids obtained from patients with active BD (1835 ± 703 U ml$^{-1}$) and from RA patients (2010 ± 504 U ml$^{-1}$).

*IL-6 secretion from alveolar macrophages from two patients with active BD:* The total BAL cell yield in BD patients (9.9 × 10$^{6}$ and 11 × 10$^{6}$ cells ml$^{-1}$) was in the same range as that of the normal group (9.7 × 10$^{6}$ cells ml$^{-1}$). BD patients had a greater percentage of lymphocytes in lavage (17% and 12%) than control population (6.8 ± 1.4%). The percentages of alveolar macrophages in the BD patients (83.4% and 79.8%) were decreased when compared to the healthy controls (93.2 ± 1.6%; $p < 0.01$).

Alveolar macrophages (AM) isolated from two patients with active BD and five controls, were assayed for spontaneous and after LPS-stimulation

IL-6 production. The level of spontaneous IL-6 production was significantly increased ($p < 0.01$) in culture supernatants from the two patients with BD, 2893 U $ml^{-1}$ and 2350 U $ml^{-1}$, as compared to supernatants of AM from five control subjects ($1200 \pm 122.5$ U $ml^{-1}$). After LPS-stimulation, AM from patients with BD produced 4540 U $ml^{-1}$ and 4980 U $ml^{-1}$, twice more than healthy controls ($2240 \pm 194.9$ U $ml^{-1}$).

Thus, spontaneous and after LPS-stimulation IL-6 production by AM from active BD patients with interstitial pneumonia was significantly increased compared to controls ($p < 0.01$).

### Discussion

In this study, a high seric IL-6 level from active BD patients similar to that from RA patients was shown, while seric IL-6 from inactive BD patients was in the same range as healthy controls. Moreover spontaneous and after LPS stimulation IL-6 production by PMBC from active BD patients, was as high as that from RA patients. Spontaneous IL-6 production from inactive BD patients was similar to that of control subjects. However after LPS stimulation, IL-6 production was as high as in active BD and RA patients.

Thus, seric IL-6 levels seemed to be correlated to the clinical activity in BD, suggesting that *in vivo*, IL-6 is produced only during the clinical exacerbations. Corticosteroid treatment may influence directly IL-6 production. It seems to downregulate *in vivo* IL-6 production in active BD, since the patients receiving steroid therapy had lower seric values. This effect was not observed with *in vitro* production of IL-6 by PMBC, since IL-6 production was high in all active BD patients, receiving or not corticosteroid therapy.

A moderate level of seric IL-6 may also be due to the presence of a circulating IL-6 receptor,[13] which diminishes IL-6 detectable activity, acting as an immunoregulator.

In the synovial fluids, IL-6 was detected at similar levels in active BD and RA patients. High IL-6 levels have been reported in several autoimmune diseases such as RA[14] and systemic lupus erythematosus.[15] IL-6 in synovial fluid from active BD was at a lower level than in RA synovial fluid. It has been reported that IL-6 production was found in inflammatory synovium from RA[14,16] and BD patients.[17]

IL-6 is an inflammatory cytokine with multiple effects including the ability to stimulate or to enhance the differentiation and the proliferation of cytotoxic T-cells,[7] and the differentiation of B-cells into plasma cells[18] as it was shown in systemic lupus erythematosus (SLE), where the overproduction of IL-6 contributes to the B-cell hyperactivity.[15] We have recently reported high cytotoxic T-cell activity against Herpes Simplex virus[19] and increased immunoglobulin production in active BD.[20] However, our data were obtained *in vitro*, and we have no evidence about the direct role of IL-6 on B-cell function.

Alveolar macrophages obtained from two BD patients with pulmonary manifestations, produced higher levels of IL-6 spontaneously and after LPS-stimulation than controls. Furthermore, AM from BD patients, in contrast to controls, expressed ICAM-1 antigen (data not shown), that could be related with the presence of IL-6. This cytokine may serve as an intermediate trigger regulating the expression of accessory surface molecules such as antigens of the MHC system and leukocyte adhesion molecules on the monocytes/macrophages.[21]

The increased IL-6 levels in serum and LPS-stimulated PBMC supernatants, did not argue against the normal levels of TNF-$\alpha$ found *in vivo*.[1] Increased concentrations of IL-1 and TNF-$\alpha$ may be found *in vivo* only in extreme circumstances,[22] whereas IL-6 is normally detected in the circulation, and rises rapidly after even minor trauma and inflammation.[23] IFN-$\gamma$ and IL-6 are known to interact either synergistically or antagonistically. In active BD, the release of gamma-interferon (IFN-$\gamma$) is increased.[4,5] It may be responsible for the enhancement of IL-6 production.[24] On the other hand, IL-6 may accelerate the inflammatory reaction by stimulating T- and B-cells, leading to further IFN-$\gamma$ production.[24]

Our aim in investigating *in vitro* and *in vivo* IL-6 production, was to study the cell–cell interactions that are influenced in a positive or a negative manner by the *in vivo* release of various mediators. An exact knowledge of the role played by each cytokine in BD, may allow to inhibit damaging cytokine pathways or amplify regulatory pathways and thereby, have a therapeutic value. Various micro-organisms have been implicated as aetiological agents of BD such as Herpes Simplex virus[25] and streptococcal antigens.[26] We are currently investigating the IL-6 and other cytokines production after stimulation with different micro-organisms.

### References

1. Hamzaoui K, Hamza M, Ayed Kh. Production of TNF$\alpha$ and IL-1 in active Behçet's disease. *J Rheumatol* 1990; **17**: 1428–1429.
2. Hamzaoui K, Ayed Kh. Soluble interleukin-2 receptors in patients with Behçet's disease. *J Rheumatol* 1989; **16**: 852–853.
3. Bacon T, Ozabakir F, Elms CEA, Denman AM. Interferon-$\gamma$ production by peripheral blood mononuclear cells from patients with Behçet's syndrome. *Clin Exp Immunol* 1984; **58**: 541–547.
4. Ohno S, Kato F, Matsuda H, Fujii N, Minagawa T. Detection of gamma-interferon in the sera of patients with Behçet's disease. *Infection & Immunity* 1982; **36**: 202–208.
5. Hamzaoui K, Ayed Kh, Slim A, Hamza M, Touraine JL. Natural killer cell activity, interferon-gamma and antibodies to herpes viruses in patients with Behçet's disease. *Clin Exp Immunol* 1990; **79**: 28–34.

6. Wong GG, Clark SC. Multiple effects of interleukin-6 within a cytokine network. *Immunol Today* 1988; **9**: 137–139.
7. Takai Y, Wong GG, Clark SC, Burakoff SJ, Herrman SH. B-cell stimulatory factor 2 is involved in the differentiation of cytotoxic T-lymphocytes. *J Immunol* 1988; **140**: 508–513.
8. Tosato G, Seamon KB, Goldman ND, *et al.* A monocyte derived human B-cell growth factor identified as interferon $\beta_2$ (BSF2, IL-6). *Science* 1988; **239**: 502–508.
9. International Study Group for Behçet's Disease (ISG). Criteria for diagnosis of Behçet's disease. *Lancet* 1990; **335**: 1078–1080.
10. Bouacha H, Azzabi S, Hamzaoui A, Kanzari A, Daoues A, Maalej M. Anévrysme de l'artère pulmonaire et infarctus pulmonaire multiples inaugurant la maladie de Behçet. *Ann Méd Interne* 1991; **142**: 544–546.
11. Hamza M. Treatment of Behçet's disease with thalidomide. *Clin Rheum* 1986; **5**: 365–371.
12. Van Damme J, Opdenakker G, Simpson RJ, *et al.* Identification of the human 26 kDa protein, interferon $\beta_2$ (IFN$\beta_2$), as a B-cell hybrodoma/plasmacytoma growth factor induced by interleukin-1 and tumour necrosis factor. *J Exp Med* 1987; **165**: 914–919.
13. Novick D, Engelman H, Wallach D, Rubinstein M. Soluble cytokine receptors are present in normal human urine. *J Exp Med* 1989; **170**: 1409–1414.
14. Houssiaux FA, Devogelaer J, Damme J, Deuxchaines CN, Snick JV. Interleukin-6 in synovial fluid and serum of patients with rheumatoid arthritis and other inflammatory arthritis. *Arthritis Rheum* 1988; **31**: 784–788.
15. Linker-Israeli M, Deans R, Wallace DD, *et al.* Elevated levels of endogenous IL-6 in systemic lupus erythematosus. A putative role in pathogenesis. *J Immunol* 1991; **147**: 117–123.
16. Miyasaka N, Sato K, Goto M, *et al.* Augmented interleukin-1 production and HLA-DR expression in the synovium of rheumatoid arthritis patients: possible involvement in joint destruction. *Arthritis Rheum* 1988; **31**: 480–486.
17. Miyasaka N, Sato K, Hashimoto J, *et al.* Constitutive production of 6/$\beta$ stimulatory factor-2 from inflammatory synovium. *Clin Immunol Immunopathol* 1989; **52**: 238–247.
18. Hirano T, Yasukawa K, Harada H, *et al.* Complementary DNA for a novel human interleukin (BSF-2) that induces B lymphocytes to produce immunoglobulin. *Nature* 1986; **324**: 73–76.
19. Hamzaoui K, Kahan A, Ayed Kh, Hamza M. Cytotoxic T-cells against herpes simplex virus in Behçet's disease. *Clin Exp Immunol* 1990; **81**: 390–395.
20. Hamzaoui K, Kahan A, Hamza M, Ayed Kh. Suppressive T-cell function of Epstein Barr virus-induced B-cell activation in active Behçet's disease. *Clin Exp Immunol* 1991; **9**: 131–135.
21. Walsh LJ, Lavker RM, Murphy GF. Determinants of immune cell trafficking the skin. *Lab Investigation* 1990; **63**: 592–600.
22. Arend W, Dayer JM. Cytokines and cytokine inhibitors or antagonists in rheumatoid arthritis. *Arthritis & Rheum* 1990; **33**: 305–315.
23. Hopkins JS. Cytokines and eicosanoids in rheumatic diseases. *Ann Rheum Dis* 1990; **49**: 207–211.
24. Leeuwenberg JFM, Von Asmuth EJU, Jeunhomme TMAA, Buurman AW. IFN$\gamma$ regulates the expression of the adhesion molecule ELMA-1 and IL-6 production by human endothelial cells *in vitro*. *J Immunol* 1990; **145**: 2110–2114.
25. Eglin RP, Lehner T, Suback-Sharpe JH. Detection of RNA complementary to herpes simplex virus in mononuclear cells from patients with Behçet's syndrome and recurrent oral ulcers. *Lancet* 1982; **ii**: 1356–1361.
26. Mizushima Y. Recent research into Behçet's disease in Japan. *Int J Tissue Reactivity* 1988; **10**: 59–63.

ACKNOWLEDGEMENTS. This study was financially supported by the 'Fondation Nationale Pour La Recherche Scientifique' of Tunisia.

**Received 12 May 1992; accepted in revised form 30 June 1992**
